# Kinesin-7 CENP-E regulates chromosome alignment and genome stability of spermatogenic cells

**DOI:** 10.1038/s41420-020-0261-8

**Published:** 2020-04-20

**Authors:** Zhen-Yu She, Kai-Wei Yu, Ning Zhong, Yu Xiao, Ya-Lan Wei, Yang Lin, Yue-Ling Li, Ming-Hui Lu

**Affiliations:** 1 Department of Cell Biology and Genetics, The School of Basic Medical Sciences, Fujian Medical University, Fuzhou, Fujian, 350122 China; 2 Key Laboratory of Stem Cell Engineering and Regenerative Medicine, Fujian Province University, Fuzhou, Fujian, 350122 China; 3 Fujian Obstetrics and Gynecology Hospital, Fuzhou, Fujian, 350001 China; 4 Medical Research Center, Fujian Provincial Children’s Hospital, Fujian Maternity and Child Health Hospital, Affiliated Hospital of Fujian Medical University, Fuzhou, Fujian, 350001 China

**Keywords:** Kinesin, Infertility

## Abstract

Kinesin-7 CENP-E is an essential kinetochore motor required for chromosome alignment and congression. However, the specific functions of CENP-E in the spermatogenic cells during spermatogenesis remain unknown. In this study, we find that CENP-E proteins are expressed in the spermatogonia, spermatocytes, and the elongating spermatids. CENP-E inhibition by specific inhibitor GSK923295 results in the disruption of spermatogenesis and cell cycle arrest of spermatogenic cells. Both spermatogonia and spermatocytes are arrested in metaphase and several chromosomes are not aligned at the equatorial plate. We find that CENP-E inhibition leads to chromosome misalignment, the spindle disorganization, and the formation of the aneuploidy cells. Furthermore, the inhibition of CENP-E results in the defects in the formation of spermatids, including the sperm head condensation and the sperm tail formation. We have revealed that kinesin-7 CENP-E is essential for chromosome alignment and genome stability of the spermatogenic cells.

## Introduction

The genome stability of eukaryotes relies on accurate segregation of chromosomes during cell division, including mitosis and meiosis^1^. Chromosome alignment and kinetochore-microtubule attachment are essential for faithful chromosome segregation^2^. Chromosome instability results in aberrant chromosome numbers (namely aneuploidy) in the daughter cells^3,4^. Defects in chromosome alignment are closely related with aneuploidy and birth defects^1^. Detail mechanisms of meiotic cell division, particularly in male mammals, remain largely unknown.

Centromere-associated protein-E (CENP-E) regulates chromosome alignment and mediates the kinetochore-microtubule attachment^5–7^. CENP-E is a member of plus-end-directed kinesin-7 subfamily, and is required for the congression of pole-proximal chromosomes^8–10^. Depletion of CENP-E in HeLa cells leads to mitotic arrest with misaligned chromosomes^11–13^. CENP-E mediates the kinetochore attachment and the tension on the centromeres^13–15^. During cell cycle, CENP-E peaks at G2/M phase and locates at the outer kinetochore plate of chromosomes in prometaphase^6,16^. CENP-E promotes kinetochore-microtubule attachment during chromosome congression^17^. CENP-E is a large kinesin motor with a 230-nm flexible coiled coil, which facilitates chromosome capture and microtubule stabilization^18–20^. CENP-E plays a role in the metaphase-to-anaphase transition. CENP-E interacts with kinetochore proteins, including Bub1, BubR1, and Mps1, to mediate spindle assembly checkpoint^21–26^.

The ablation of CENP-E in HeLa cells leads to chromosome misalignment with mono-oriented chromosomes at the spindle poles^13,27,28^. Deletion/inhibition of CENP-E results in metaphase arrest with several misaligned chromosomes and a prolonged cell cycle^13^. Deletion of *CENP-E* in mice shows chromosome missegregation and early embryo death^5,26^. CENP-E heterozygous (*CENP-E*^*+/−*^) mice show increased aneuploidy and tumor formation^4,29^.

Genetic deletion of CENP-E^5,26^, the absence of CENP-E at kinetochores^14^, and the inhibition of CENP-E’s motor activity^18,30^ suggest that CENP-E is essential for the faithful chromosome segregation. CENP-E regulates the chromosome alignment and spindle assembly checkpoint during meiosis II^1,31^. In *D*. *melanogaster* oocytes, CENP-E is essential for the alignment and movements of homologous chromosomes at meiosis I^32^. However, the specific functions of CENP-E in male meiotic division remain obscure.

The small molecule GSK923295 is a specific allosteric inhibitor of CENP-E, which inhibits the release of inorganic phosphate and stabilizes CENP-E in a rigor microtubule-bound state^33–35^. GSK923295 treatment results in mitotic arrest and chromosome misalignment in metaphase. In tumor tissues, the ratio of 4N to 2N nucleus increases significantly after GSK923295 treatment. GSK923295-medieated CENP-E inhibition results in chromosome misalignment, cell cycle arrest, apoptosis, and tumor regression^35^.

In this study, we have revealed the expression pattern of kinesin-7 CENP-E in mouse spermatogenic cells. CENP-E proteins are expressed in the spermatogonia and spermatocytes. CENP-E locates at the manchette of the elongating spermatids during spermatogenesis. We have found that CENP-E inhibition results in the disruptions in spermatogenic waves and metaphase arrest of the spermatogium and spermatocytes. The ablation of CENP-E leads to chromosome misalignment in spermatocytes both in vivo and in vitro, which then stimulates the formation of aneuploidy cells. CENP-E regulates chromosome alignment in meiosis of primary spermatocyte. In addition, we have revealed that CENP-E inhibition influences nuclear condensation and the structures of sperm flagellum. In summary, we have revealed that kinesin-7 CENP-E plays an essential role in chromosome alignment and spindle assembly in spermatocytes, which promotes chromosome integrity and genome stability of male gametes.

## Results

### CENP-E proteins are expressed in the spermatogenic cells and CENP-E inhibition disrupts the normal cycles of spermatogenesis

**Fig. 1:**
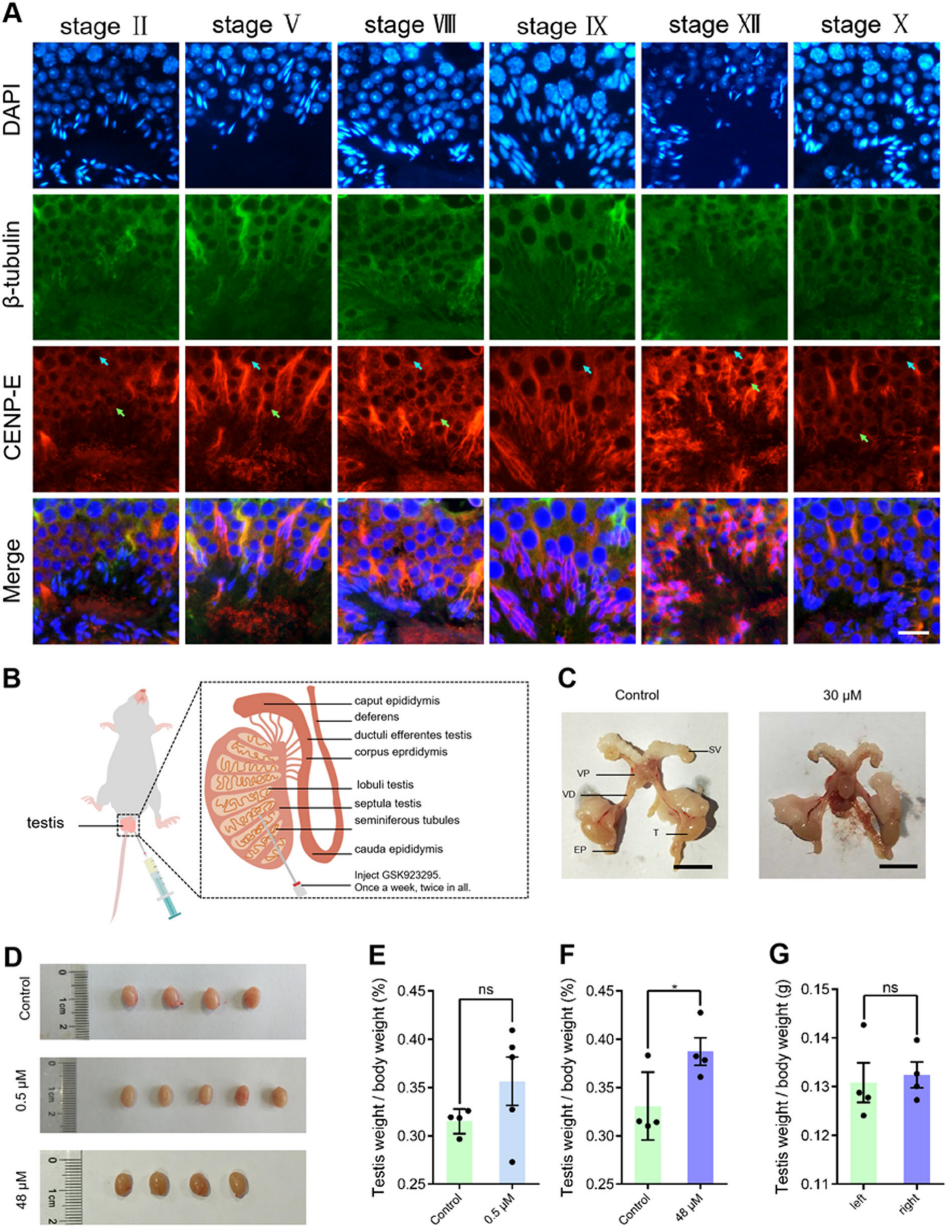
The expression pattern of kinesin-7 CENP-E in mouse spermatogenic cells. **a** Immunofluorescence of CENP-E in mouse spermatogenic cells in testicular seminiferous tubules. DAPI was used to stain the nucleus. DAPI (blue), β-tubulin (green), and CENP-E (red). **b** Construction of a mouse model. Different concentrations of GSK923295 (14 μM) were injected into the left testis of 8-week-old ICR mice to achieve inhibition of CENP-E. **c** Representative images of the male mouse reproductive system. Testis (T), epididymis (EP), seminal vesicles (SV), ventral prostate (VP), and vas deferens (VD). Scale bar, 1 cm. **d** Representative images of mouse testes injected with 0.5 μM, 30 μM GSK923295. *n* = 4 or *n* = 5 per group. Control: 0.79 ± 0.03 cm; 0.5 μM: 0.82 ± 0.02 cm; 30 μM: 0.78 ± 0.02 cm. **e**–**g** Ratios of testis weight/body weight of testes after injected with 0.5 μM and 30 μM GSK923295. Mean values ± SEM were shown. ns, *p* > 0.05, *p < 0.05, **p < 0.01 and ***p < 0.001. See also Fig. S1

To study the expression pattern of CENP-E proteins in mouse spermatogenic cells, we examined the localization of CENP-E proteins in mouse testes using immunofluorescence (Figs. 1; S1). CENP-E proteins were expressed in the spermatogonia, spermatocytes, and elongating spermatids. CENP-E proteins located at the cytoplasm in spermatogenic cells (Fig. 1a). At stage II, CENP-E proteins were distributed in the spermatogonia. At stage V, CENP-E signals reached peak in the step 15 elongating spermatids. At stage IX, CENP-E located at the manchette of step 9 spermatids. In the elongating spermatids, CENP-E located at the microtubules of manchette (Fig. 1a). Thus, CENP-E proteins are expressed in all spermatogenic cells, indicating that CENP-E may play a role in spermatogenesis.

To investigate the functions of CENP-E in male mouse gonads, we used a small molecule inhibitor GSK923295, which specifically inhibited the ATP release of CENP-E and locked CENP-E in a rigor microtubule-bound state^34,35^ (Fig. 1b). We injected the GSK923295 into the right testis of 6-week-old ICR mice at a final concentration of 0.5, 1, 2, 4, and 30 μM, respectively (Fig. S1a, b). We compared the reproductive systems in the control and the GSK923295 group (Fig. 1b, c). There was no difference in the long diameter of mouse testes (Fig. 1d). The ratios of testis weight/body weight among 0.5, 1, 2, and 4 μM GSK923295 treated group were similar to the control (Fig. 1e, f). After 30 μM GSK923295 inhibition, the testis weight increased slightly compared with the control (Fig. 1g). Taken together, GSK923295 is a useful tool for long-term inhibition of CENP-E in vivo. And CENP-E inhibition does not significantly influence the weight of mouse testes.

**Fig. 2:**
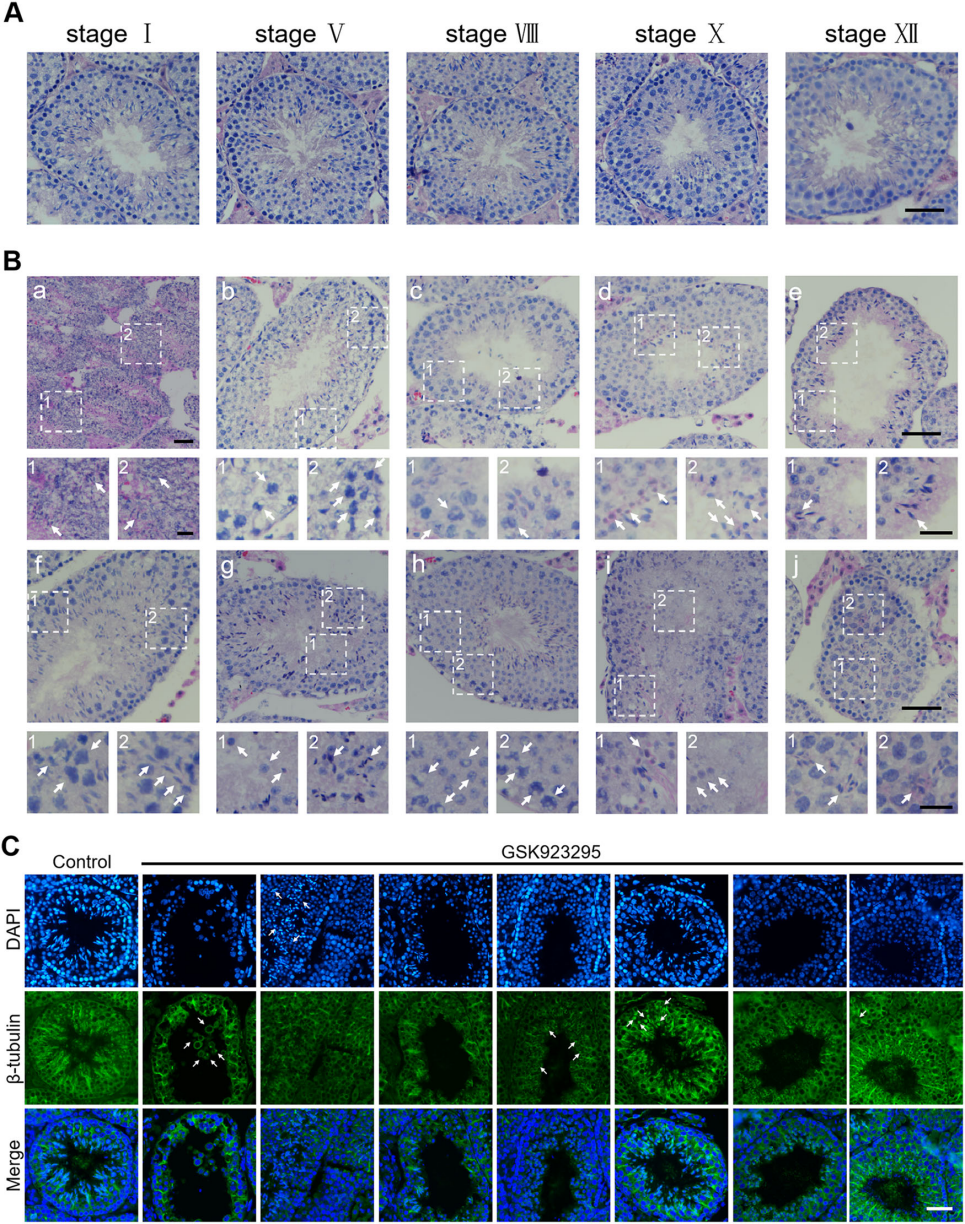
CENP-E inhibition results in the testicular structural disorder and metaphase arrest of spermatocytes. **a**, **b** Representative images of HE staining of the mouse testicular sections in the control (A) and GSK923295 (B) group. Scale bar, 50 μm. Relative stages were shown at the top of the panel. The enlarged images of spermatogenic cells were shown at the bottom of the panel. Scale bar, 20 μm. The arrows indicate the abnormal spermatogonia (c1), the arrested spermatocytes (b1, b2), and the dead sperms (d1). **c** Representative images of immunofluorescence of the mouse testicular sections. DAPI (blue) and β-tubulin (green). Scale bar, 50 μm. See also Fig. S2

To further study the effects of CENP-E inhibition in spermatogenesis at the cellular level, we examined the cellular structure of spermatogenic cells in seminiferous tubules using HE staining. In control, all spermatogenic cells were arranged orderly in spermatogenic epithelium. The spermatogenic epithelial waves were normal (Fig. 2a). We found that seminiferous tubules of the GSK923295 group were disordered. The thickness of each seminiferous tubule became smaller. The arrangement of spermatogonia and spermatocytes in the seminiferous tubules were abnormal. Specific stages of spermatogenic epithelial waves could not be clearly staged (Figs. 2b, c; S1c). In several seminiferous tubules, sperm cells were severely deformed and the head of the sperms were usually round. The spermatogenic epithelium became thinner. The distribution of spermatids was scattered and a portion of spermatids resided inside the seminiferous tubules (Fig. 2b, c). In addition, several spermatogenic cells showed a vacuolar structure, which might be lipid droplets observed by transmission electron microscopy (Fig. S1d).

### CENP-E inhibition results in the metaphase arrest of the spermatocytes during meiosis I

At the cellular level, we found that CENP-E inhibition led to various abnormalities in spermatogenic cells. After GSK923295 treatment, a portion of the spermatogonia were arrested in metaphase during mitosis. Several chromosomes failed to align at the equatorial plate in metaphase. Notably, we found that a significantly increased number of primary spermatocytes were arrested in metaphase I. In GSK923295 treated spermatocytes, several chromosomes were not aligned at the equatorial plate in metaphase (Fig. 2b). After CENP-E inhibition for 2 weeks, metaphase-arrested spermatocytes were a common phenotype. The primary spermatocytes were arrested in metaphase I with misaligned chromosomes away from the equatorial plate. These results indicate that CENP-E regulates chromosome alignment and cell cycle progression of primary spermatocytes.

**Fig. 3:**
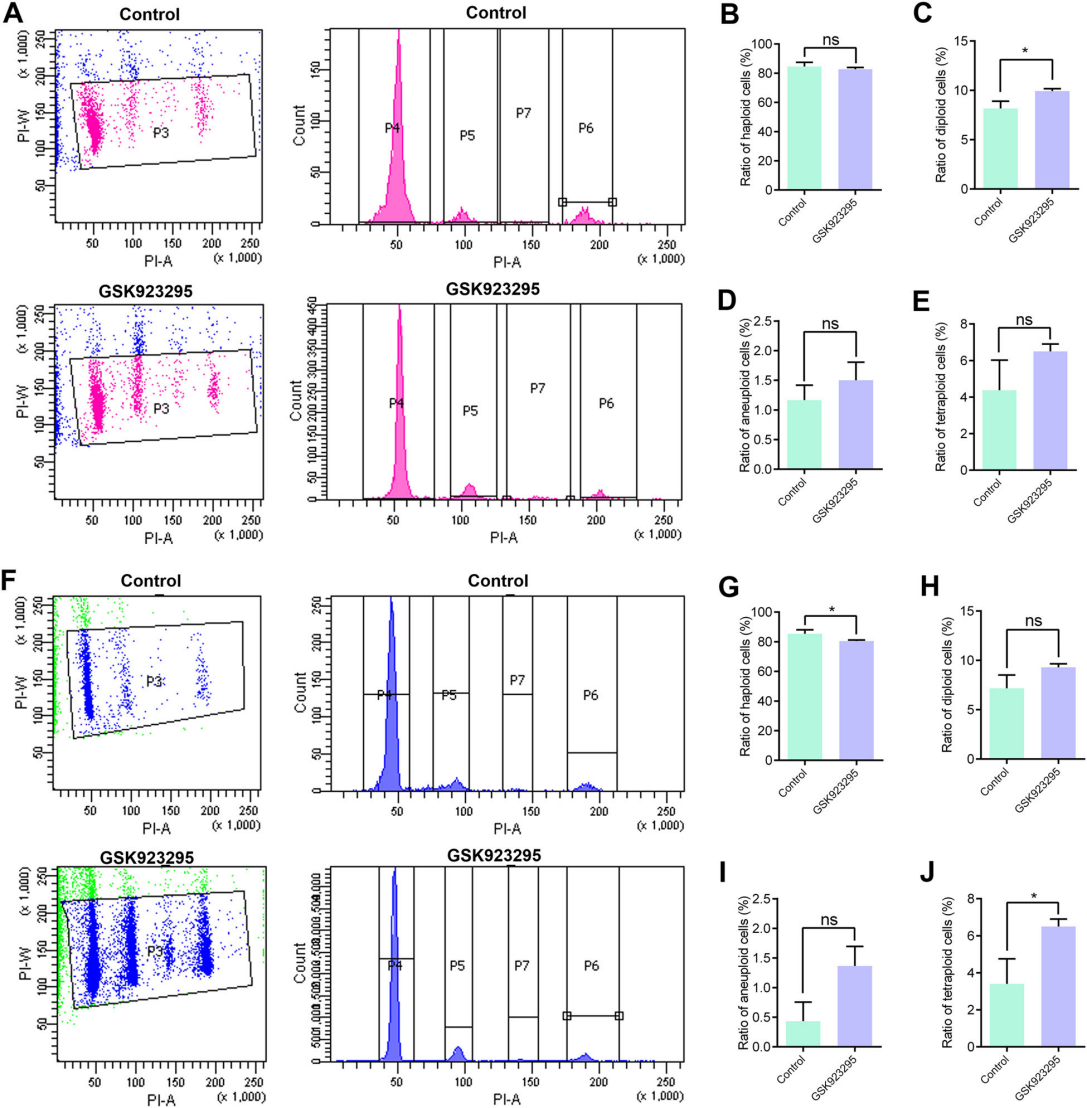
Cell cycle analyses of the spermatogenic cells in mouse testes. **a** Flow cytometry of the spermatogenic cells in mouse gonads in the control and GSK923295 group. GSK923295 was injected five times every 3 days at a final concentration at 30 μM (Control: *n* = 1937; GSK923295 group, *n* = 3156). **b**–**e** The ratios of the haploid cells (P4: Control, 84.57 ± 1.65%; GSK923295, 82.73 ± 1.18%), the diploid cells (P5: Control, 8.17 ± 0.43%; GSK923295, 9.93 ± 0.23%), the aneuploidy cells (P7: Control, 1.17 ± 0.15%; GSK923295, 1.50 ± 0.31%) and the tetraploid cells (P6: Control, 4.38 ± 0.82%; GSK923295, 6.50 ± 0.40%) in mouse testes. **f** Flow cytometry of the spermatogenic cells in mouse gonads in the control and GSK923295 group. GSK923295 was injected by intraperitoneal injection for 10 times every 2 days at a final concentration of 30 μM (Control, *n* = 2056; GSK923295 group, *n* = 25648). **g**–**j** The ratios of the haploid cells (P4: control, 85.35 ± 1.36%; GSK923295, 80.48 ± 0.70%), the diploid cells (P5: Control, 7.18 ± 0.68%; GSK923295, 9.3 ± 0.36%), the aneuploidy cells (P7: Control, 0.43 ± 0.19%; GSK923295, 1.37 ± 0.33%) and the tetraploid cells (P6: Control, 3.40 ± 0.78%; GSK923295, 6.50 ± 0.40%). ns, *p* > 0.05, *p < 0.05, **p < 0.01, and ***p < 0.001

To further investigate the cell populations in mouse testes, we digested the testes and stained the cells with propidium iodide (PI) for flow cytometry. In control, all the spermatogenic cells were divided into four groups according to the amounts of DNA content, including the haploid cells (spermatids and sperms, 1N), the diploid cells (spermatogonia and primary spermatocytes, 2N), the tetraploid cells (dividing primary spermatocytes, 4N), and a small portion of aneuploidy cell (2–4N). After the injection of GSK923295 into mouse testes for 2 weeks, the ratios of the diploid cells increased from 8.17% to 9.93% in mouse testes (Fig. 3a–e). These results indicate that an increase in the ratios of spermatogonia and primary spermatocytes.

To study the long-term effects of GSK923295 inhibition, we injected GSK923295 into the abdomen of mice. In the intraperitoneal injection group for 10 times every 2 days, we observed a decrease in the proportion of haploid cells and a significant increase in tetraploid cells (Fig. 3f–j). These results suggest that the long-term inhibition of CENP-E leads to a decrease in the numbers of spermatids and dividing primary spermatocytes (Fig. 3). We also found that the number of spermatogenic cells between 2 and 4N (aneuploidy cells) increased significantly. In the peak diagram, tetraploid cells also increased significantly (Fig. 3f), which indicated that cell cycle arrest and tetraploid cells could not divide normally. Taken together, the long-term inhibition of CENP-E leads to the metaphase arrest of primary spermatocytes and the formation of aneuploidy cells during spermatogenesis.

**Fig. 4:**
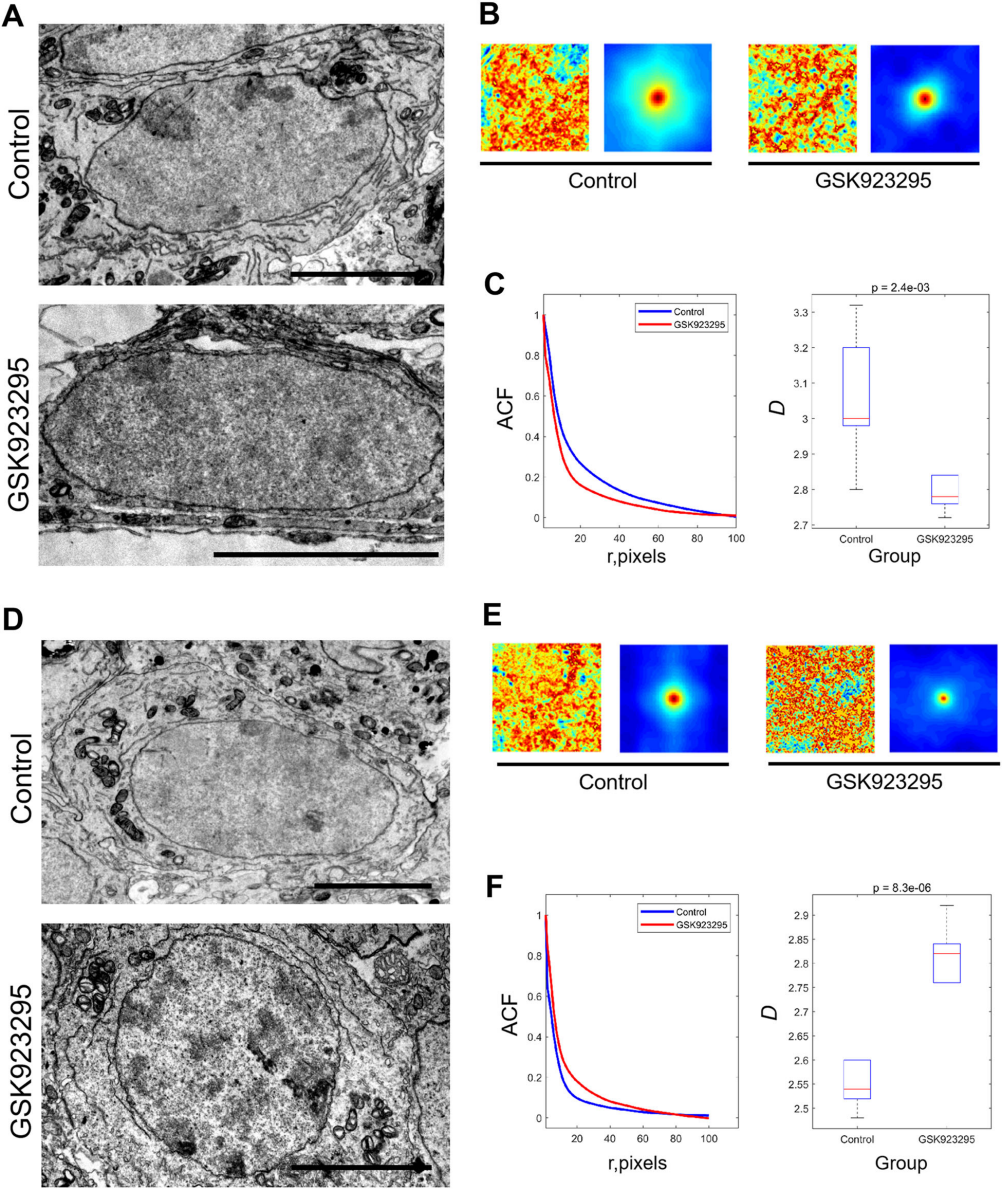
Electron microscopic analysis of mouse spermatogenic cells. **a** Representative images of electron micrograph of mouse spermatogonia in the control and GSK923295 group. Scale bar, 5 μm. **b** The origin analyzed figures and two-dimensional autocorrelation heat map for the measurement of mass density correlation function of the spermatogonia in the control and GSK923295 group (*n* = 6). **c** The average ACF (autocorrelation functions) and a boxplot of calculated *D* values in the control and GSK923295 treated spermatogonia. The ACF and *D* value represent the morphology of the chromatin mass density distribution. The *D* value is a parameter describing the ACF based on the Whittle-Matern family of functions^55^. **d** Representative images of electron micrographs of mouse spermatocytes in the control and GSK923295 group. Scale bar, 5 μm. **e** The origin analyzed figures and two-dimensional autocorrelation heat map for the measurement of mass density correlation function of the spermatogonia in the control and GSK923295 group (*n* = 6). **f** Comparison of the spatial ACF and relative *D* values in the control and GSK923295 treated spermatocytes. The boxplots show all values of *D* in the correlation functions. See also Fig. S5

We found that the nuclei of spermatogonia and spermatocytes after CENP-E inhibition were slightly different from the control group (Fig. 4). The quantifications of chromatin mass density indicated that the heterochromatin increased in the nucleus of spermatogonia and spermatocytes (Fig. 4a–c, e, f). In spermatocytes, the organization of the nucleolus, including the fibrillar center, the dense fibrillary component and granular component, was slightly influenced after CENP-E inhibition (Fig. 4d–f).

### CENP-E inhibition leads to chromosome misalignment and spindle defects in dividing spermatocytes

**Fig. 5:**
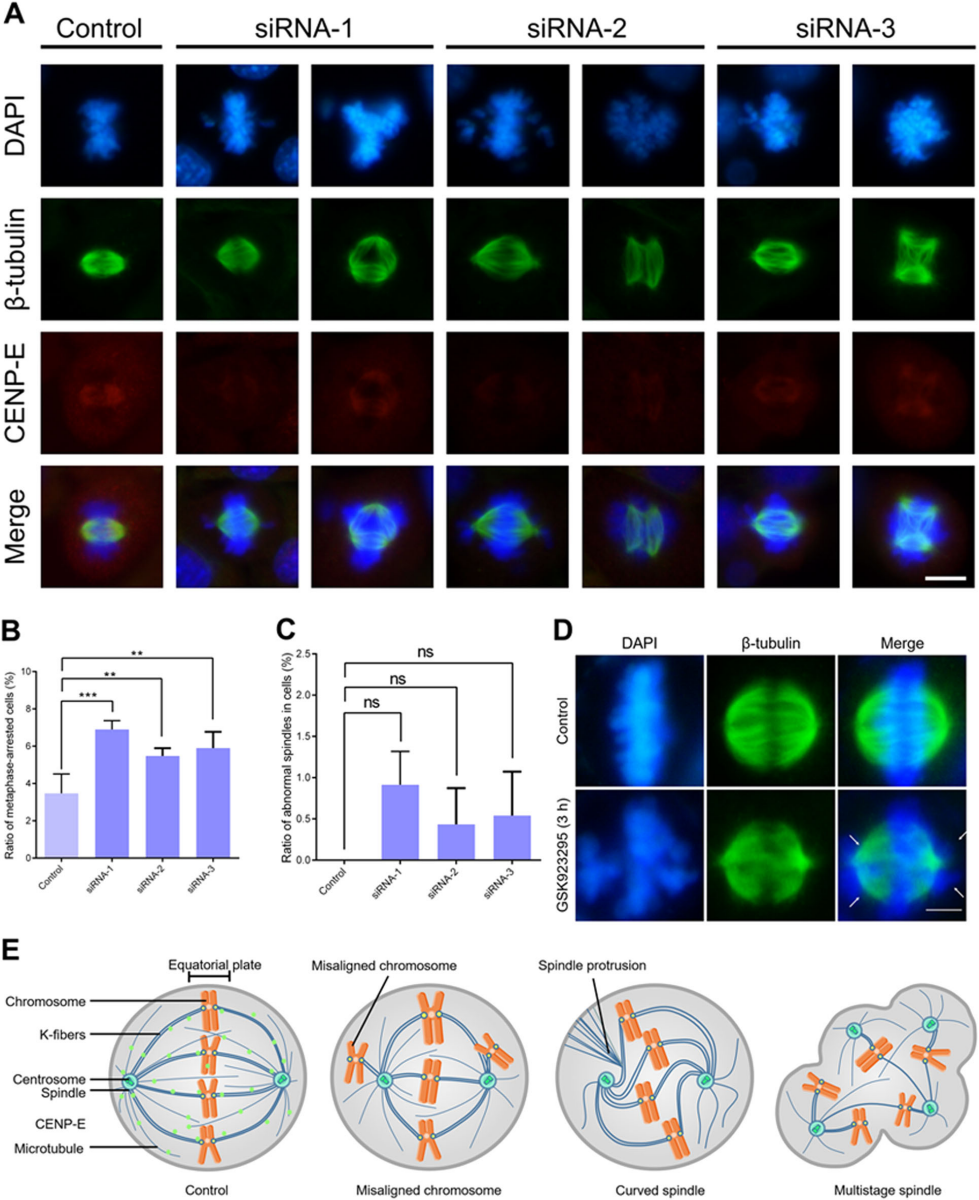
CENP-E regulates chromosome alignment in the GC-2 spd cells. **a** Representative images of the GC-2 spd cells in the control and siRNA groups. Three different siRNA targeting CENP-E were transfected into the GC-2 spd cells and then cultured for 24 h. DAPI (blue), β-tubulin (green), and CENP-E (red). Scale bar, 10 μm. **b** Ratios of metaphase-arrested cells in the control group and the siRNA knockdown groups. Control: 3.47 ± 0.46%; siRNA-1: 6.90 ± 0.47%; siRNA-2: 5.48 ± 0.18%; siRNA-3: 5.90 ± 0.39%; *n* = 692, 681, 779, 611; group = 5. **c** Ratios of abnormal spindles in the control group and the siRNA knockdown groups. Control: 0.00 ± 0.00%; siRNA-1: 0.91 ± 0.40%; siRNA-2: 0.43 ± 0.20%; siRNA-3: 0.54 ± 0.24%; *n* = 692, 681, 779, 611; group = 5. **d** Representative images of HeLa cells in the control and 400 nM GSK923295 groups. The misaligned chromosomes were shown. DAPI (blue) and β-tubulin (green). Scale bar, 10 μm. **e** The model of normal cells and CENP-E depleted cells were shown. The ablation of CENP-E resulted in chromosome misalignment in dividing spermatocytes and HeLa cells

To study the underlying causes of chromosomal aneuploidy and cellular abnormalities, we firstly selected the GC-2 spd cells as our model cells. The GC-2 spd (ts) cell line was established by stable transfection of mouse spermatocytes with the SV40 large T antigen gene. The GC-2 spd cells remained at the spermatocyte stage and could not differentiate^36^. Thus, we selected the GC-2 spd cell as our model cell line to study cell division of spermatocytes in vitro. We treated the GC-2 spd cells with 100 nM siRNA and then cultured for 24 and 48 h. We found that CENP-E knockdown resulted in chromosome misalignment in the GC-2 spd cells at metaphase (Fig. 5a). In 6% GC-2 spd cells, the ablation of CENP-E led to several chromosomes can not align at the equatorial plate (Fig. 5b). We also compared the spindle abnormalities in CENP-E-depleted GC-2 spd cells with the control group. We found that there was no significant difference in the control and siRNA groups (Fig. 5c). After CENP-E knockdown for 24 h, a significantly increased number of cells showed chromosome misalignment and metaphase arrest (Fig. 5a–c).

We also treated HeLa cells with GSK923295 and then cultured for 3, 6, or 24 h. After GSK923295 treatment for 3 h, we observed that several chromosomes could not align at the equatorial plate and a portion of HeLa cells were arrested in metaphase (Fig. S3). The inhibition of CENP-E resulted in 82.06% of HeLa cells became round and arrested at metaphase compared with 7.20% in the control (Fig. S3a, c). Giemsa staining showed that GSK923295 treatment resulted in a decrease in the number of HeLa cells, indicating that CENP-E inhibition led to a longer duration of cell cycle (Fig. S3b). After inhibition of CENP-E, a number of HeLa cells showed metaphase arrest. In addition, there were abnormalities in huge cells and multinucleated cells (Fig. S3d, e). In addition, we also found several abnormal spindle microtubule disorganization in HeLa cells, including the monopolar spindles, the multipolar spindles, the spindle protrusion and the curved spindles (Fig. S3f, i). These results indicate that CENP-E is essential for chromosome alignment in both cultured spermatocytes and HeLa cells.

**Fig. 6:**
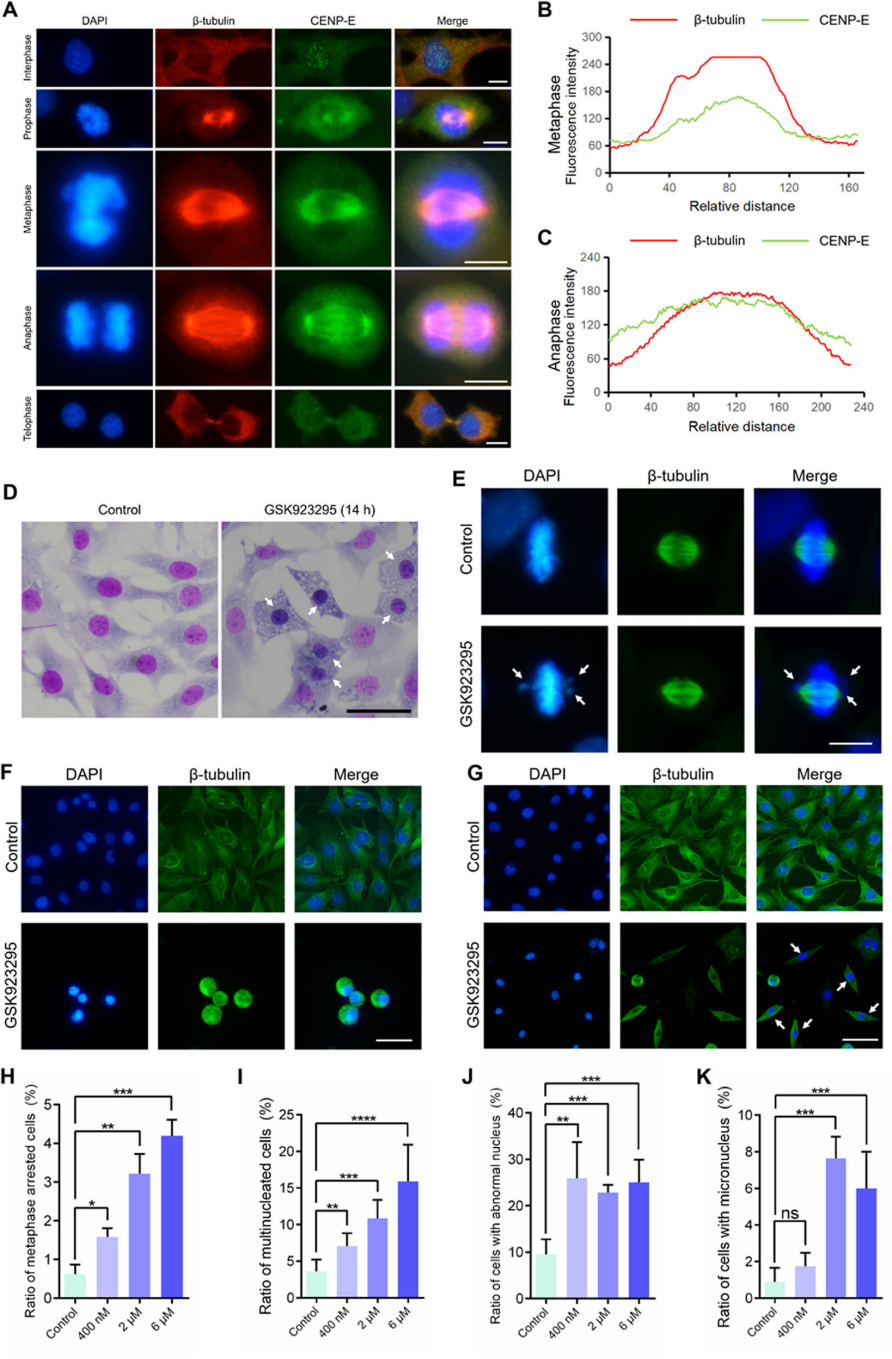
CENP-E inhibition results in spindle disorganization and chromosome misalignment in the GC-2 spd cells. **a** Immunofluorescence of CENP-E in the GC-2 spd cells. DAPI (blue), CENP-E (green), and β-tubulin (red). Scale bar, 10 μm. **b**, **c** Image J analyses of the immunofluorescence intensities of indicated lines in GC-2 spd cells in metaphase and anaphase. **d** Giemsa staining of the GC-2 spd cells after incubated with 400 nM GSK923295 for 14 h. **e** Immunofluorescence of the GC-2 spd cells after incubated with 100 nM siCENP-E-3 for 24 h. DAPI (blue) and β-tubulin (green). Scale bar, 10 μm. **f**, **g** Immunofluorescence of the GC-2 spd cells after incubated with 400 nM GSK923295 for 14 h. DAPI (blue) and β-tubulin (green). Scale bar, 50 μm. **h** The ratios of metaphase arrested cells (**h** Control, 0.61 ± 0.25%, group = 5, *n* = 747; 400 nM, 1.58 ± 0.22%, group = 5, *n* = 630; 2 μM, 3.22 ± 0.51%, group = 3, *n* = 364; 6 μM, 4.20 ± 0.42%, group = 506, *n* = 3). **i** The ratios of the multinucleated cells (**i**; Control, 9.50 ± 1.63%, group = 4, *n* = 691; 400 nM, 25.94 ± 3.16%, group = 6, *n* = 300; 2 μM, 22.83 ± 0.83%, group = 4, *n* = 595; 6 μM, 25.04 ± 2.18%, group = 5, *n* = 488). **j** The ratios of cells with abnormal nucleus (**j** Control, 3.60 ± 0.57%, group = 4, *n* = 691; 400 nM, 7.07 ± 0.78%, group = 6, *n* = 300; 2 μM, 10.84 ± 1.262%, group = 4, *n* = 595; 6 μM, 15.88 ± 2.25%, group = 5, *n* = 488). **k** The ratios of cells with micronuclei (**k** Control, 0.89 ± 0.34%, group = 4, *n* = 691; 400 nM, 6.00 ± 0.90%, group = 6, *n* = 300; 2 μM, 1.74 ± 0.33%, group = 4, *n* = 595; 6 μM, 7.64 ± 0.59%, group = 5, *n* = 488). See also Fig. S3

Kinesin-7 CENP-E proteins located at the regions of centromeres in the nucleus of the GC-2 spd cells in interphase (Fig. 6a). In prophase, CENP-E proteins located at the cytoplasm and spindle microtubules. In metaphase, all chromosomes were aligned at the equatorial plate and a portion of CENP-E proteins located at kinetochores (Fig. 6a–c). In anaphase, CENP-E proteins located at central spindle and the cytoplasm (Fig. 6a, c). To investigate specific roles of CENP-E in dividing spermatocytes, we examined the morphology of the GC-2 spd cells after GSK923295 treatment. Interestingly, we found that the staining of the nucleus in the GC-2 spd cells significantly changed compared with the control after Giemsa staining (Fig. 6d). Meanwhile, vacuolization abnormalities in the GC-2 spd cells were common after CENP-E inhibition (Fig. 6d). We found that a portion of chromosomes were not aligned at the equatorial plate after the siRNA-mediated knockdown of CENP-E in the GC-2 spd cells (Fig. 6e). In addition, a portion of GC-2 spd cells were round in interphase (Fig. 6f). The morphology of the GC-2 spd cells changed from flatten to fusiform (Fig. 6g).

We found that CENP-E inhibition resulted in several phenotypes, including chromosome misalignment in the metaphase arrested cells (Fig. 6h), the multinucleated cells (Fig. 6i), the abnormal nucleus (Fig. 6j), and the formation of micronuclei (Fig. 6k). We found that ratios of metaphase arrested GC-2 spd cells significantly increased to 1.58–4.20% in the presence of 400 nM, 2 μM, and 6 μM GSK923295 compared with 0.61% in the control (Fig. 6h). The ratios of multinucleated cells also significantly increased to 22.83–25.94% in the GSK923295 treated group compared with 9.50% in the control (Fig. 6i). Taken together, CENP-E inhibition results in metaphase arrest of dividing spermatocytes in vitro. The multinucleated cells and cells with micronuclei also increased after GSK923295 treatment, indicating that CENP-E inhibition led to the failure in chromosome segregation.

### CENP-E inhibition results in sperm head malformation and principal piece bending

**Fig. 7:**
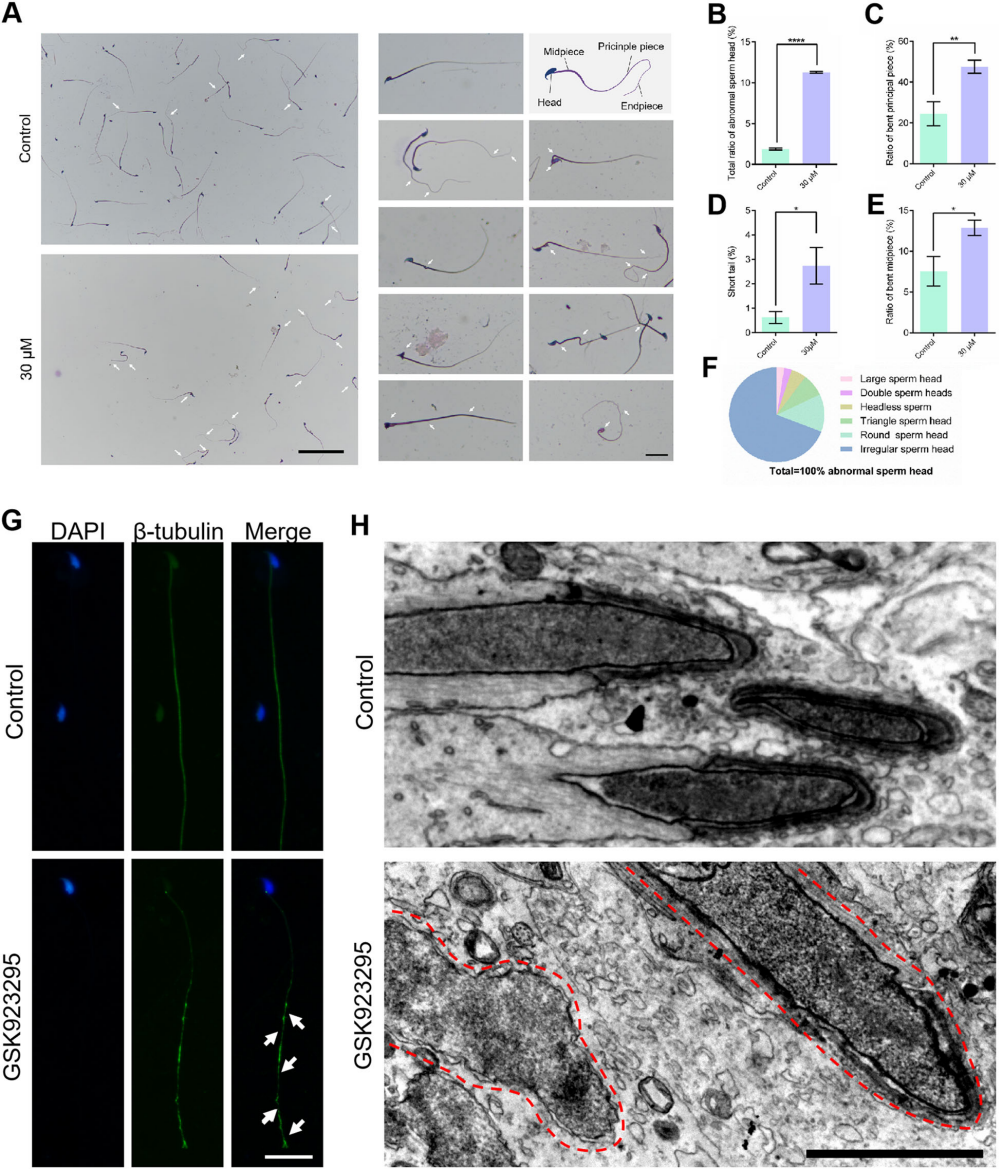
CENP-E inhibition leads to the abnormality in the sperm nucleus and tail formation. **a** HE staining of the semen smear in the control and GSK923295 (30 μM) group. Scale bar, 100 μm. The enlarged images of the sperms were shown in the left panel. Scale bar, 20 μm. **b**–**e** The ratios of abnormal sperm head (control, 1.90 ± 0.07%; GSK923295, 11.29 ± 0.11%), the bent principal piece (control, 24.47 ± 3.42%; GSK923295, 47.55 ± 3.20%), the short tail (control, 0.62 ± 0.14%; GSK923295, 2.74 ± 0.75%), and the bent midpiece (control, 7.53 ± 1.05%; GSK923295, 12.86 ± 0.94%). **f** The ratios of the irregular sperm head, the round sperm head, the triangle sperm head, the headless sperm. Control, group = 3, *n* = 635; GSK923295, group = 3, *n* = 691. **g** Immunofluorescence of the sperms. Scale bar, 20 μm. **h** TEM images of the sperms. Scale bar, 2 μm. See also Fig. S4

In order to study whether CENP-E inhibition influences the morphology and structure of mature spermatozoa, we squeezed the semen and stained the spermatozoa using HE staining (Fig. 7a). The abnormal sperms in the GSK923295 treated group significantly increased compared with the control. There were four kinds of sperm deformities, including the abnormal sperm head (Fig. 7b), the bent principal piece (Fig. 7c), the short tail malformation (Fig. 7d), and the bent midpiece (Fig. 7e). The ratios of these four kinds of malformation in sperms increased significantly compared with the control (Fig. 7b–e).

The long-term inhibition of CENP-E resulted in severe deformity of the mature spermatozoa in mice after the injection of GSK923295 for 2 weeks (Figs. 7; S4). We divided the sperm head malformations into six subtypes, including 69.23% of the irregular head sperms, 12.82% of the round head sperms, 7.69% of the triangular head sperms, 5.12% of the headless sperms, 2.56% of the large head sperms, and 2.56% of the double head sperms (Fig. 7f). These results indicate that CENP-E inhibition affects the morphology of the sperm head, the midpiece, the principal piece, and the endpiece.

We labeled β-tubulin to detect the structures of microtubules in the spermatozoa after CENP-E inhibition. We found that the microtubules of the flagella became discontinuous (Fig. 7g). Using electron microscopy, we found that the sperm head was relatively regular in the control group, but the nuclear shape was deformed and electron density of the nucleus was abnormal in GSK923295 group (Fig. 7h). In addition, the spermatids and spermatocytes in the control group had uniform nucleus, which contained homogenous chromatin (Fig. S5). However, CENP-E inhibition resulted in the irregularly distributed heterochromatin in the nucleus in spermatids and spermatocytes (Fig. S5b).

### Acute treatment of GSK923295 affects sperm maturation and causes sperm abnormality

**Fig. 8:**
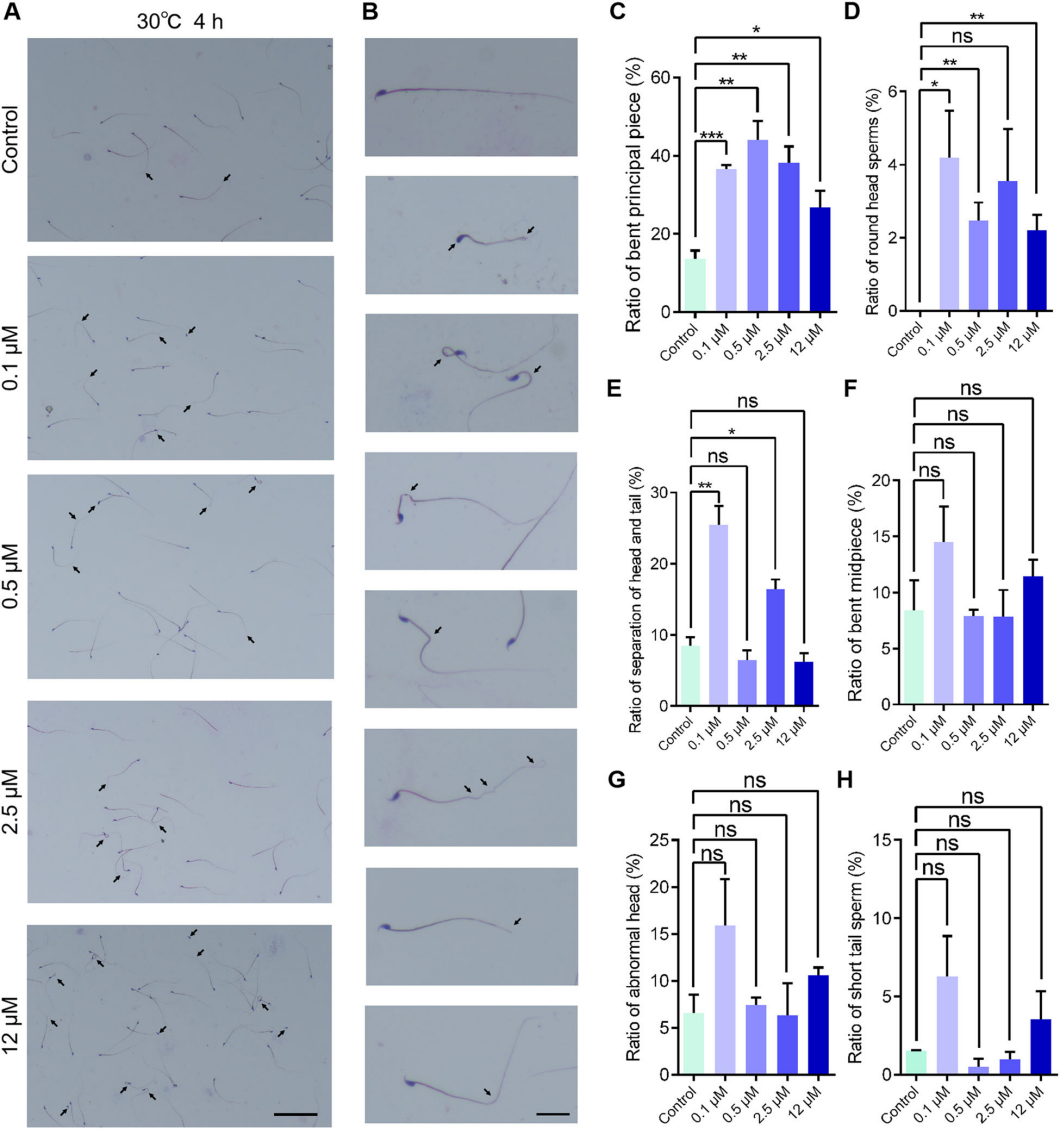
CENP-E inhibition results in abnormal sperms. **a** HE staining of the semen smear in the control and GSK923295 (0.1 μM, 0.5 μM, 2.5 μM, and 12 μM) group. Scale bar, 100 μm. **b** The enlarged images of the sperm in the control and GSK923295 group. Scale bar, 20 μm. The types of abnormality in sperms includes: knotting of sperm head, round sperm head, bending of sperm midpiece, bending of sperm endpiece, and shortening of sperm tail. **c**–**h** The analysis of ratio of sperms’ abnormality in the control and GSK923295 (0.1 μM, 0.5 μM, 2.5 μM and 12 μM) group. The *Y* axis means bending of sperm endpiece (**c** control, 13.68 ± 2.01%; 0.1 μM, 36.55 ± 0.98%; 0.5 μM, 44.09 ± 4.82%; 2.5 μM, 38.27 ± 4.09%; 12 μM, 26.80 ± 4.20%), round sperm head (**d** control, 0.0 ± 0.0%; 0.1 μM, 4.19 ± 1.30%; 0.5 μM, 2.47 ± 0.49%; 2.5 μM, 3.56 ± 1.42%; 12 μM, 2.20 ± 0.43%), tailless sperm (**e** Control, 8.50 ± 1.20%; 0.1 μM, 25.45 ± 2.66%; 0.5 μM, 6.46 ± 1.37%; 2.5 μM, 16.48 ± 1.29%; 12 μM, 6.19 ± 1.21%), knotting of sperm head (**f** control, 8.40 ± 2.67%; 0.1 μM, 14.52 ± 3.18%; 0.5 μM, 7.93 ± 0.52%; 2.5 μM, 7.86 ± 2.37%; 12 μM, 11.43 ± 1.48%), bending of sperm midpiece (**g** control, 6.58 ± 1.93%; 0.1 μM, 15.90 ± 4.94%; 0.5 μM, 7.41 ± 0.79%; 2.5 μM, 6.35 ± 3.38;12 μM, 10.59 ± 0.84%), shortening of sperm tail (**h** control, 1.52 ± 0.06%; 0.1 μM, 6.30 ± 2.55%; 0.5 μM, 0.51 ± 0.51%; 2.5 μM, 0.97 ± 0.49%; 12 μM, 3.56 ± 1.78%), respectively. The *X* axis means the concentration of GSK923295 (0.1 μM, 0.5 μM, 2.5 μM and 12 μM). Control, group = 3, *n* = 199; 0.1 μM, group = 3, *n* = 145; 0.5 μM, group = 3, *n* = 202; 2.5 μM, group = 3, *n* = 201; 12 μM, group = 3, *n* = 227. The mean ± SEM were shown. The significance of differences (*p* > 0.05, ns; *p < 0.05, **p < 0.01, ***p < 0.001, ****p < 0.0001) was analyzed by unpaired Student’s *t* test

In order to illustrate whether acute treatment of GSK923295 influences the morphology of the mature spermatozoa, we performed a short-term inhibition of CENP-E in the mature spermatozoa. We aimed to find out the effects of CENP-E inhibition at the mature sperm stage. The epididymis of 6-month-old male mice was extracted and diluted with 0.1, 0.5, 2.5, and 12 μM GSK923295, respectively. The semen was cultured at 30 °C for 4 h in vitro (Fig. 8). The results showed that in the acute GSK923295 treatment group, the number of spermatozoa with the bent principal piece increased to 26.80–44.09% in the GSK923295 treated group compared with 13.68% in the control group (Fig. 8a–c). The round head sperms also significantly increased (Fig. 8d). In contrast, there was no significant difference in the number and proportion of spermatozoa among the separation of the head and tail, the bent midpiece, the abnormal head, and the short tail sperm (Fig. 8e–h).

These results suggest that short-term inhibition of CENP-E also leads to abnormal structure of sperm tail and a small number of round-headed spermatozoa. However, the short-term inhibition of CENP-E results in a less deformity of sperm head compared with the long-term treated group. This indicates CENP-E involves in all the process of spermatogenesis, including the formation of nucleus at early stage and maintenance of spermatozoa in the mature stage.

In addition, we examined apoptosis of spermatogenic cells after GSK923295 treatment using the TUNEL assays (Fig. S6). We did not observe a large number of the apoptotic cells in cultured HeLa cells and the GC-2 spd cells (Fig. S6a), and in seminiferous tubules after 2 weeks inhibition (Fig. S6b). These results indicate that GSK923295 treatment does not result in the apoptosis of spermatogenic cells.

## Discussion

### Kinesin-7 CENP-E is expressed in mouse spermatogenic cells

CENP-E proteins locate at the centromeres in prometaphase of the cultured spermatocytes. In metaphase, CENP-E proteins locate at kinetochore fibers in dividing spermatocytes. During cytokinesis, CENP-E proteins locate at the central spindle and the midbody. In seminiferous tubules, CENP-E proteins are expressed in the spermatogenic cells (Fig. 1).

In human cells, CENP-E locates at the fibrous corona and the outer kinetochore plate^37^. In our study, we reveal that expression pattern of CENP-E in spermatocytes is similar to the previous results in somatic cells^7,37,38^, indicating a conserved role of CENP-E in mitosis and meiosis. CENP-E proteins are initially located at the outer kinetochore plate of homologous centromeres in late dikinesis/early prometaphase I during male mouse meiosis^1,31,39^. The inner centromere proteins MCAK and SGOL2 recruit CENP-E and BubR1 to the outer kinetochore plate of primary spermatocytes^1^. However, the specific functions of CENP-E in male mouse meiotic divisions remain largely unknown.

Our results indicate that CENP-E plays a role in male meiotic division. The small molecule inhibitor GSK923295 inhibits the ATPase activity of CENP-E and locks the motor domain in a rigor microtubule-bound state^35^. Thus, GSK923295 is a unique tool to investigate the long-term consequence of CENP-E inhibition and developmental functions of CENP-E in this study.

### CENP-E is essential for the metaphase-to-anaphase transition in primary spermatocytes

In somatic cells, CENP-E inhibition results in metaphase arrest and mitotic delay^6^. However, there are significant differences in the process of meiosis I and mitosis. For example, in meiosis I, homologous chromosomes undergo recombination and separation. However, in mitosis, sister chromatids separate and the cohesion breakdown^40^. Meiotic spindles also differ from the typical mitotic spindle^27^. Most studies of CENP-E are performed in somatic cells, while little information is available regarding the functions of CENP-E in mammalian meiosis and male spermatogenesis. In our study, we have revealed that CENP-E inhibition results in metaphase arrest of primary spermatocytes in meiosis I. This is the first evidence that CENP-E also involves in the regulation of the metaphase-to-anaphase transition of meiosis I.

Anti-CENP-E antibody injection into maturing mouse oocytes results in metaphase I arrest and metaphase II arrest^40^, indicating that CENP-E might have a conserved role in female meiosis division. In our study, we have found that CENP-E inhibition by GSK923295 can result in the metaphase I arrest of primary spermatocytes both in vivo and in vitro. These results indicate that CENP-E is responsible for the metaphase-to-anaphase transition of primary spermatocytes in meiosis I.

In prometaphase, the unattached kinetochores produce a “wait anaphase” signal for the activation of spindle assembly checkpoint^23^. CENP-E interacts with the checkpoint components, including BubR1 and Mad2^11,41^, and then participates in the establishment and maintenance of the spindle assembly checkpoint^21,23^. Microtubule capture by CENP-E prevents the recruitment of Mad2 to kinetochores under tension and silences the BubR1-dependent spindle assembly checkpoint^3,25,42^. Spindle assembly checkpoint is weakened at CENP-E-depleted kinetochores and a portion of chromosomes can not form the bipolar attachment^25,27,43^. In HeLa cells, siRNA-mediated CENP-E inhibition leads to a delayed mitotic progression for 4 h due to misaligned chromosomes^27,43^. In our study, we have found that the metaphase arrested spermatocytes significantly increased both in vitro and in vivo (Fig. 2). Meanwhile, several chromosomes misaligned at the spindle poles. Whether metaphase arrest in meiosis I is caused by the activation of spindle assembly checkpoint remains to be clarified in future.

### CENP-E regulates chromosome alignment and spindle organization in spermatocytes

Depletion of CENP-E leads to chromosome misalignment in *Xenopus* egg extracts^27^, *D*. *melanogaster*^44^, and vertebrate cultured cells^12^. CENP-E is a plus-end-directed kinetochore motor essential for chromosome alignment at the metaphase plate^27,45^. Kinetochore-microtubule interaction is regulated by CENP-E, which is crucial for chromosome alignment in the equator plate^12^. The ablation of CENP-E leads to significant numbers of mono-oriented chromosomes at the spindle poles^12,14^. CENP-E transports mono-oriented chromosomes to the spindle equator along mature kinetochore fibers^46,47^, and then stabilizes metaphase alignment^48^. CENP-E is a highly processive motor that generates forces^49^ and tethers the kinetochore to dynamic spindle microtubules^8,19^. Similarly, we have found that CENP-E inhibition results in chromosome misalignment in dividing spermatocytes (Fig. 6). These results suggest that CENP-E is also required for chromosome alignment in meiosis.

Chromosome missegreagtion and cytokinetic defects are caused by the defects in spindle assembly^50^. Previous studies have indicated that CENP-E depletion reduce kinetochore-microtubule attachment and tension^14,51^. The depletion of CENP-E leads to 50% decrease in the density of the kinetochore fibers^5,14^. In our study, we have found that CENP-E inhibition results in multiple spindle defects, including curved spindles and microtubule disorganization in both HeLa and the GC-2 spd cells. In addition, CENP-E contains a 230-nm flexible coiled coil, which is involved in microtubule capture^19^. The elongated stalk domain is essential for the stabilization of kinetochore-microtubule attachment^52^. In mouse oocyte meiosis I, CENP-E depletion results in the polar chromosome displacement and the unstable kinetochore-microtubule attachment^53^. CENP-E stabilizes BubR1 to promote meiosis I progression and regulates the chromosome bi-orientation by inhibition of chromosome drift to the spindle poles^53^. After chromosome congression, CENP-E severs as a processive bi-directional tracker for stable association between dynamic microtubule ends and kinetochores^18^. In male mouse meiosis, the misaligned chromosome at the spindle poles might be due to the microtubule capture by CENP-E is inhibited.

### CENP-E is responsible for genome stability of spermatogenic cells and the formation of sperms

The stable propagation of genetic material relies on the chromosome congression at the spindle equator^46^. Genetic deletion of CENP-E in mice results in misaligned chromosomes in primary fibroblasts and high degree of aneuploidy, even to embryonic lethal^5^. The primary cultured cells and hepatocytes only proceed through several rounds of mitotic division^5^. CENP-E deletion reduces the number of kinetochore-associated microtubules, which leads to the lack of kinetochore-microtubule attachment^5^.

Heterozygous deletion of CENP-E in mice leads to gain or loss of intact chromosomes, but does not result in cytokinesis failure and tetraploidy^29^. In CENP-E deficient cells, the sister chromatids can not separate normally, which then increase the whole chromosome aneuploidy^54^. In our study, we have found that CENP-E inhibition in dividing spermatocytes leads to the increase of multinucleated cells and cells with micronuclei (Fig. 4). These results suggest that the gain and loss of intact chromosomes also exist in cell division of spermatocytes after CENP-E inhibition, which increases aneuploidy in daughter cells.

The inhibitor GSK923295 is reported to cause apoptosis in human HCC1954 breast carcinoma cells^35^. In our study, the TUNEL results indicate that few apoptotic cells exist and an increased numbers of dead cells after GSK923295 treatment. Previous studies have indicated that high chromosomal instability leads to cell death^4^. The increased number of dead cells in seminiferous tubules is caused by the missegreagtion of high numbers of chromosomes. In addition, abnormal structures and irregular shapes of the sperm midpiece, principal piece, and endpiece are mainly caused by microtubule instability after CENP-E inhibition during the spermatogenesis. We have found that both spermatogenesis and mature sperms are affected by the inhibition of CENP-E.

Our results address a long-standing question in cell division. Previous studies have demonstrated that CENP-E regulates chromosome congression and alignment in somatic cells and mediates spindle assembly checkpoint. In summary, our data provide the initial evidence that CENP-E is essential for chromosome alignment and congression in meiosis. CENP-E inhibition results in the defects in chromosome misalignment and congression, which leads to metaphase arrest in meiosis. CENP-E regulates the organization of the bipolar spindle to fulfill accurate chromosome alignment. CENP-E inhibition results in chromosome misalignment, chromosome missegreagtion, and the formation of aneuploidy cells. In addition, the depletion of CENP-E results in the defects in the spermatid formation, including the sperm head condensation and the sperm tail formation. Taken together, we have concluded that kinesin-7 CENP-E regulates the chromosome alignment in meiosis and maintains the genome stability of male spermatids.

## Materials and methods

### Animals and ethics

All animal experiments were conducted according to the Guide for the Care and Use of Laboratory Animals of Fujian Medical University. This study was approved by the Animal Care and Use Committee at Fujian Medical University (Protocol No. SYXK 2016-0007).

### Drugs and reagents

GSK923295 (MedChemExpress HY-10299) was dissolved in dimethyl sulfoxide (DMSO, Sigma-Aldrich D2650) and stored at a final concentration of 10 mM. Different concentrations of GSK923295 were diluted in H_2_O or 0.9% NaCl for CENP-E inhibition. For RNA interference in mouse GC-2 spd cells, the following siRNAs were synthesized: Negative Control, 5′-UUCUCCGAACGUGUCACGUTTT-3′; si*Gapdh*, 5′-GUAUGACAACAGCCUCAAGTT-3′ (*Mus musculus Gapdh*, GenBank Accession No. NM_001289726.1); si*CENP-E*-1, 5′-GGAAGAAAGUCAAGAGGAATT-3′; si*CENP-E*-2, 5′-CUGCUGAACUGGAGAGAAATT-3′; si*CENP-E*-3, 5′-UGAAAGAGCAGGAGAACAATT-3′ (*Mus musculus CENP-E*, GenBank Accession No. NM_173762.4).

### Cell culture and treatment

The GC-2 spd cell (ATCC No. CRL-2196) and HeLa cell (ATCC No. CCL-2) were cultured in DMEM/high glucose medium (Hyclone, Cat. SH30022.01) supplemented with 10% fetal bovine serum (Every green, Cat. 11011-8611), 4 mM l-glutamine (Hyclone), 4.50 g/L glucose (Hyclone), and 100 IU/mL Penicillin-Streptomycin (MP Biomedicals, Cat. 1670249). The cell lines was authenticated and tested for mycoplasma contamination. The identity of all cell lines are verified annually in our laboratory. The cells were cultured at 37 °C and supplemented with 5% CO_2_ in a humidified incubator (Thermo Fisher). For cell passage, 0.25% Trypsin-EDTA (Gibco Cat. 25200-056) was used for digest. For CENP-E inhibition in cultured cells, GSK923295 were added into the culture medium as indicated and then cultured at 37 °C. For CENP-E siRNA interference, the siRNA, including negative control, si*Gapdh*, si*CENP-E*-1, si*CENP-E*-2, and si*CENP-E*-3, was transfected into the GC-2 spd cells using Lipo 8000 transfection reagent according to the manufacturer’s protocol (Beyotime Cat. C0533). The siRNA were incubated with Lipo8000 for 5 min and added into the culture medium at a final concentration at 100 μM. The GC-2 spd cells were then cultured for 24 and 48 h and harvested for subsequent analyses.

### Cryo-section and immunofluorescence

The 8-week-old male ICR mice were killed according to the standard protocols of animal experiments of Fujian Medical University. The testes or epididymis were collected and measured. The testes and epididymis were incubated with 4% polyformaldehyde/PBS solution for 12 h and then dehydrated in 20% sucrose/PBS for 4 h, and then in 30% sucrose/PBS for 4 h. The samples were dissected into small pieces and embedded in Tissue-Tek O.C.T. compound (Sakura Cat. 4583). After incubated with liquid nitrogen for 2 min, the samples were stored at −80 °C for 12 h. The 6-μm cryo-sections were cut using a Leica freezing microtome (Leica CM1860 UV) and then stored at −80 °C for subsequent analyses.

The cryo-sections were fixed with 4% PFA/PBS for 10 min, and then immersed in PBS solution three times for 3 min. For permeation, the slides were incubated with 0.5% TritonX-100/PBS at room temperature for 10 min. The slides were blocked with 1% BSA/PBST (0.05% Tween 20 in PBS) for 1 h. The specific primary antibody was diluted in 1% BSA/PBST and incubated with the samples at 4 °C for 12 h. After washed by PBS for 15 min, the samples were incubated with the secondary antibody at 37 °C for 1 h. 4′, 6-diamidino-2-phenylindole (DAPI) (Beyotime Cat. C1006) were used for the staining of the nucleus. The slides were incubated with the anti-fading medium (Beyotime Cat. P1028S) and observed under a fluorescence microscope (Nikon Ti-S). The antibodies used in this study are listed as follows: the CENP-E mouse monoclonal antibody (Santa Cruz Cat. SC-376685, 1:100), β-tubulin rabbit monoclonal antibody (Beyotime Cat. AF1216, 1:500), Alexa Fluor 488-labeled Goat Anti-Rabbit IgG (H + L) (Beyotime Cat. A0423, 1:500), Alexa Fluor 488-labeled Goat Anti-Mouse IgG (H + L) (Beyotime Cat. A0428, 1:500), Alexa Fluor 555-labeled Donkey Anti-Rabbit IgG (H + L) (Beyotime Cat. A0453, 1:500), and Alexa Fluor 555-labeled Donkey Anti-Mouse IgG (H + L) (Beyotime Cat. A0460, 1:500).

### Hematoxylin-eosin staining

For Hematoxylin-eosin (HE) staining of testis cryo-sections, the slides were incubated with 70% ethanol for 2 min, and then incubated with distilled water for 2 min. After incubating with Mayer’s hematoxylin solution for 5 min, the slides were washed by running water for 5 min. The samples were incubated with saturated lithium carbonate for 5 s, and then incubated with distilled water for 2 min. After incubating with 1% eosin for 1 min, the samples were dehydrated by gradient ethanol and cleared in xylene for 5 min. The samples were fixed with neutral resin for observation.

For HE staining of semen smear, semen was squeezed out of the epididymis, and then diluted using 0.9% NaCl. GSK923295 was added into 0.9% NaCl to the final concentration as indicated. The sperms and GSK923295 were mixed and then incubated at 30 °C for 4 h. The slides were dried at room temperature for 10 min. After incubating with 95% ethanol for 10 min, the slides were rinsed in distilled water for 3 min. After incubating with Mayer’s hematoxylin for 5 min, the samples were incubated with ethanol hydrochloride for 30 s. After staining by eosin for 1 min, the samples were fixed by 95% ethanol for 5 min. The slides were photographed by a light microscope (Nikon Ti–S) with a NA ×20/0.40 objective and a NA ×40/0.75 objective (Nikon).

### Flow cytometry

For cell cycle analysis of spermatogenic cells, the testes were cut and digested with trypsin (Beyotime Cat. C0201) at 37 °C for 15 min. After washed twice in PBS for 3 min, the cells were incubated with 75% ethanol for 12 h. The cells were suspended in PBS and pelleted by centrifugation at 1200 × *g*. The samples were incubated with propidium iodide (Beyotime Cat. C1052) at 37 °C for 2 h. The flow cytometry was performed using a flow cytometer (BD, FACS Canto TM II).

### TUNEL assay

For cell apoptosis analysis, the one step TUNEL apoptosis assay kit (Beyotime Cat. C1086) was used. The cells were fixed with 4% paraformaldehyde/PBS for 1 h. After washing twice with PBS for 10 min, the samples were incubated with 0.5% Triton X-100 in PBS for 5 min at room temperature. The samples were incubated with TdT solution (terminal deoxynucleotidyl transferase (TdT) and fluorescein isothiocyanate (FITC)-dUTP, 1:10) at 37 °C for 1 h. After washing three times with PBS, the slides were mounted with anti-fading medium and observed using a fluorescence microscope (Nikon Ti–S).

### Transmission electron microscopy

For sample fixation: 3% glutaraldehyde, 1.5% polyformaldehyde in 0.1 M PBS (pH7.4) was used. After rinsing with 0.1 M PBS, the samples were fixed using 1% osmium solution. After rinsing with 0.1 M PBS, the samples were dehydrated using 30–90% gradient alcohol, 90–100% acetone, and then 100% acetone epoxy resin (v/v, 1:1) at room temperature for 2 h. After incubating with epoxy resin at 35 °C for 3 h, the samples were incubated with epoxy resin at 35 °C for 12 h, at 45 °C for 12 h, at 60 °C for 3 days. In all, 100 nm ultra-thin section were cut using an ultra-thin slicing machine (Leica EM UC-7). The samples were stained with uranium acetate for 20 min. After rinsing with distilled water, the samples were stained using lead citrate for 5 min. After rinsing with distilled water, the samples were imaged using an electron microscope (FEI, Tecnai G2). The quantification of chromatin arrangement and density was performed using a script from github (https://github.com/barouxlab/ChromDensityNano) by the MATLAB software according to previous reported methods^55^.

### Image analyses and statistics

All experiments were performed more than three times. The sample size was indicated in each figure legend. The investigator was blinded to the group allocation during the experiment and/or when assessing the outcome. The simple randomization was used in the sample analysis and animal experiments. Statistical analyses were conducted by the two-tailed unpaired Student’s *t* test using GraphPad Prism 6.0 software. Data were shown as mean ± SEM. ns, *p* > 0.05, *p < 0.05, **p < 0.01, ***p < 0.001, and ****p < 0.0001.

## Supplementary information

Figure S1

Figure S2

Figure S3

Figure S4

Figure S5

Figure S6

Supplementary Figure Legends

## Data Availability

The data that support the findings of this study are available from the corresponding author upon reasonable request.
