# Supplementary figures and images for: Kinesin-7 CENP-E regulates chromosome alignment and genome stability of spermatogenic cells
Source: Cell Death Discov. 2020 Apr 20;6:25. doi: 10.1038/s41420-020-0261-8 (PMC7171076; doi:10.1038/s41420-020-0261-8)

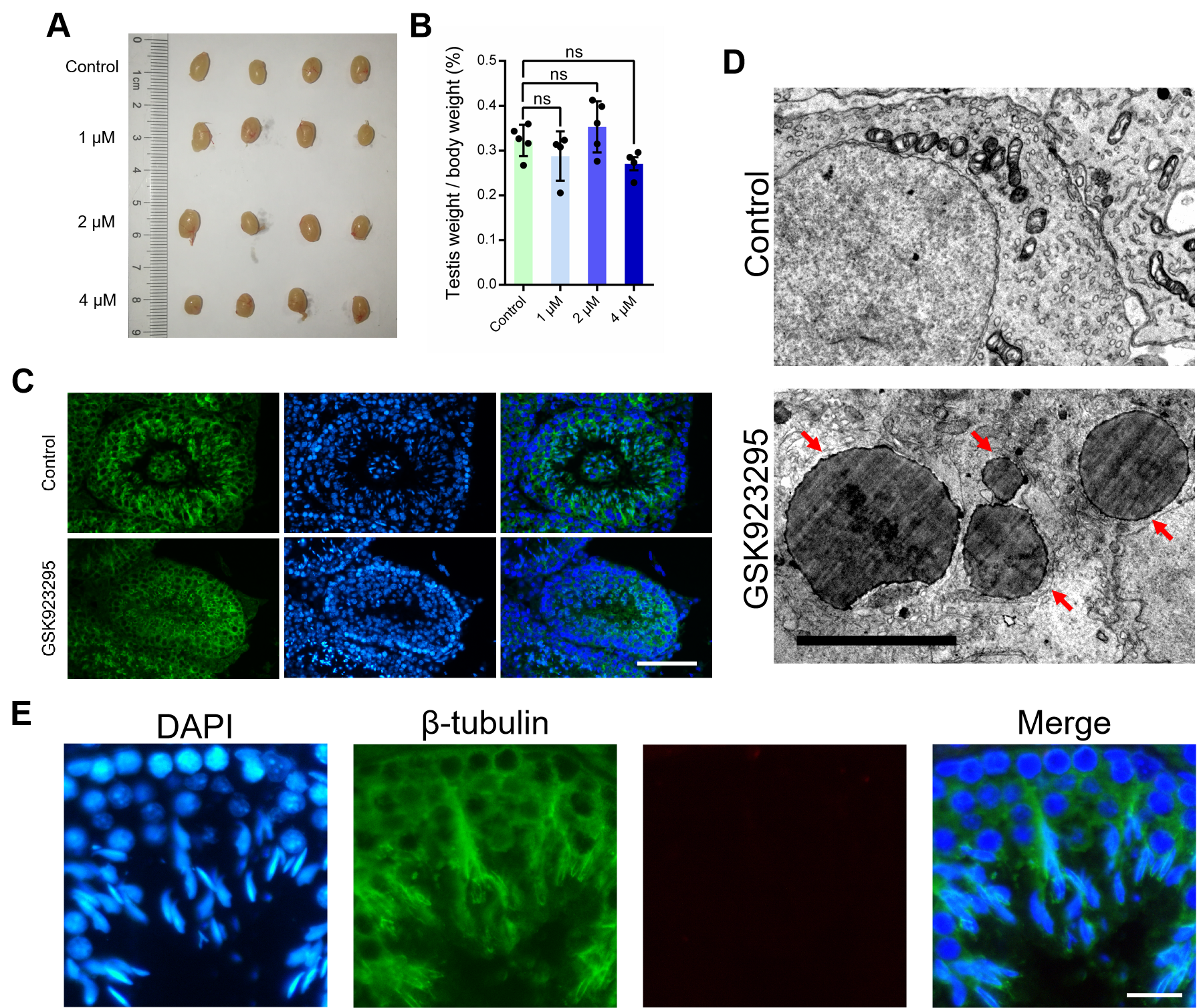

Supplement: Supplementary file 1 — Figure S1 [file 41420_2020_261_MOESM1_ESM.tif]

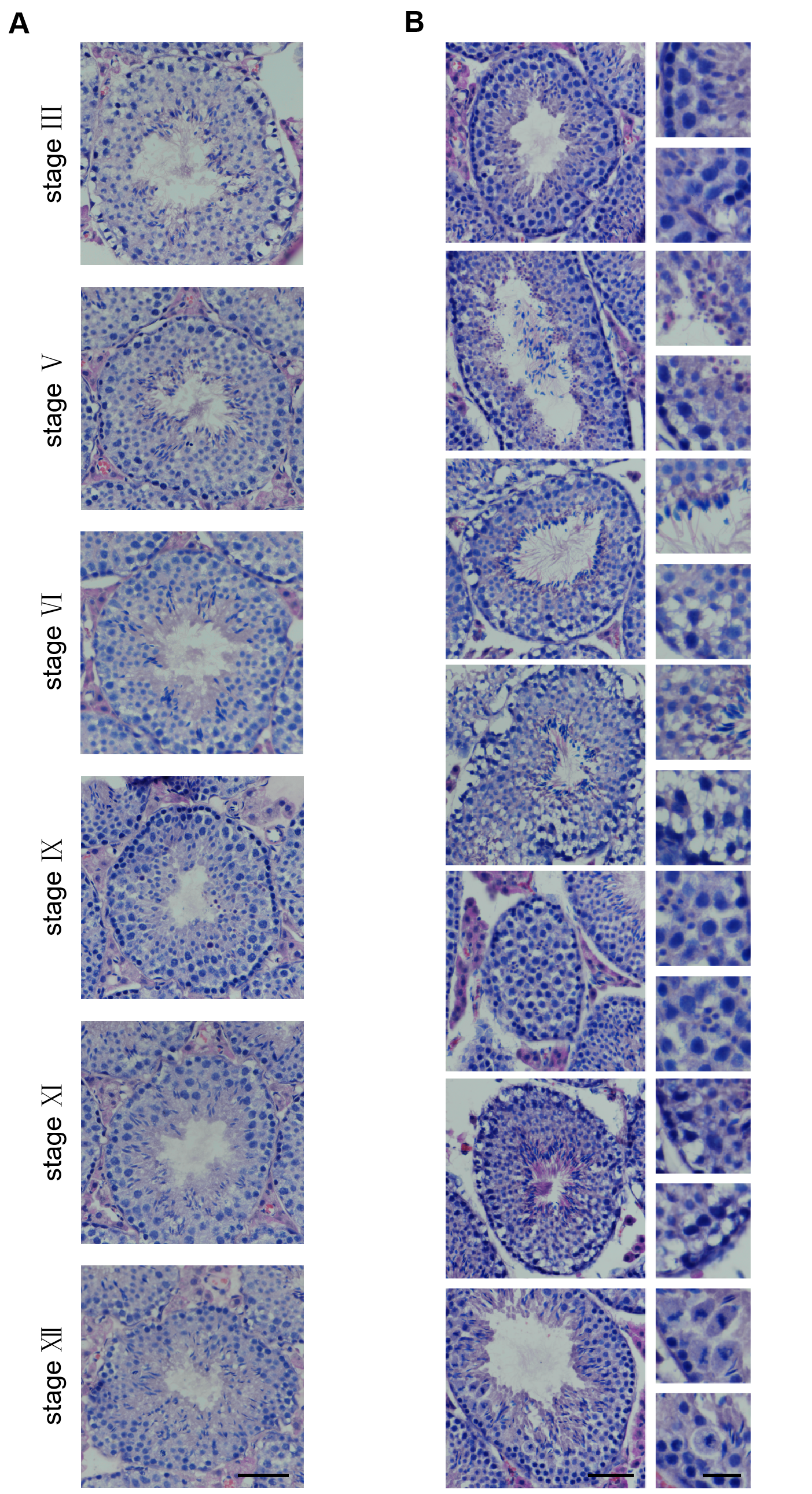

Supplement: Supplementary file 2 — Figure S2 [file 41420_2020_261_MOESM2_ESM.tif]

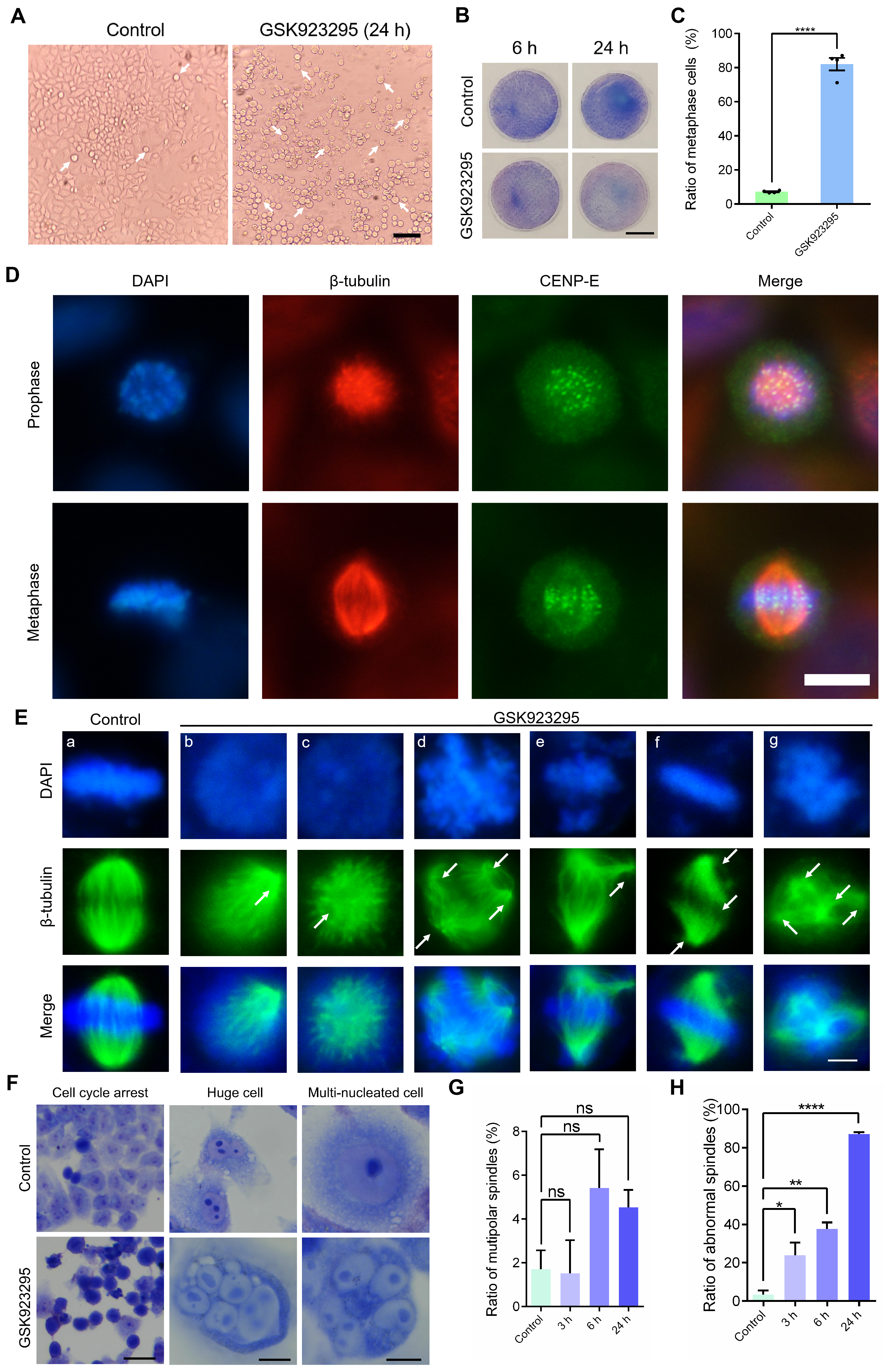

Supplement: Supplementary file 3 — Figure S3 [file 41420_2020_261_MOESM3_ESM.tif]

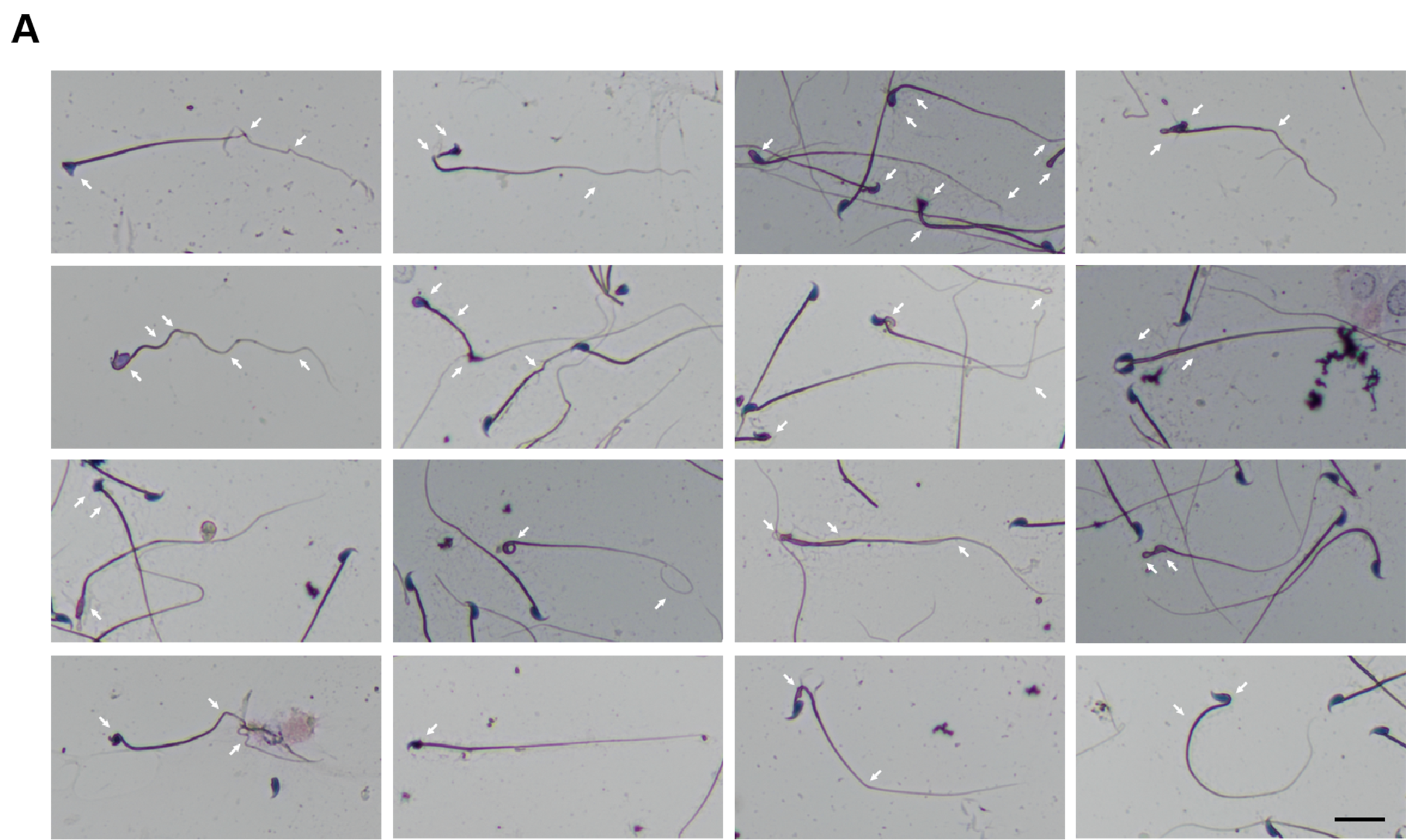

Supplement: Supplementary file 4 — Figure S4 [file 41420_2020_261_MOESM4_ESM.tif]

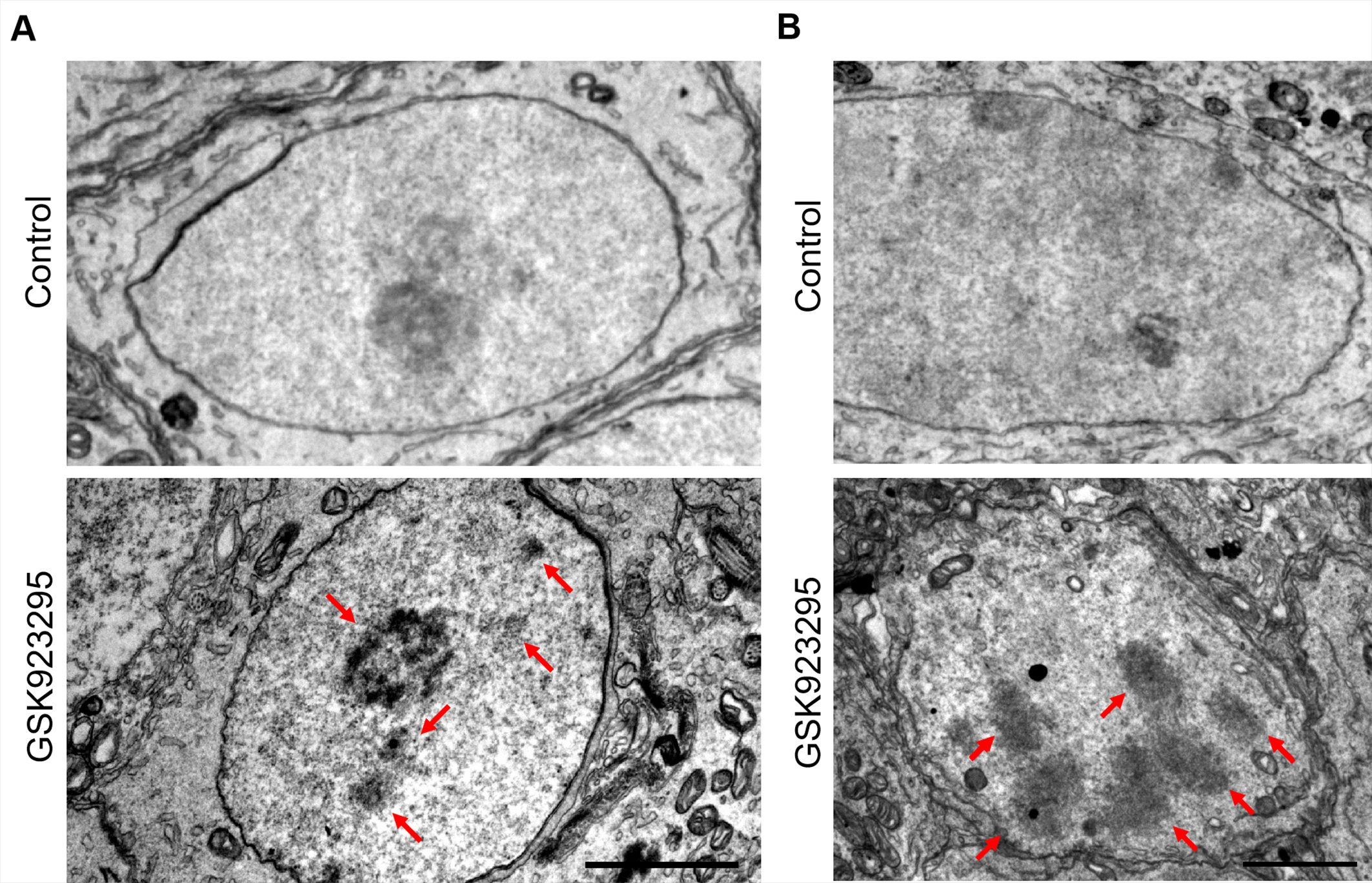

Supplement: Supplementary file 5 — Figure S5 [file 41420_2020_261_MOESM5_ESM.tif]

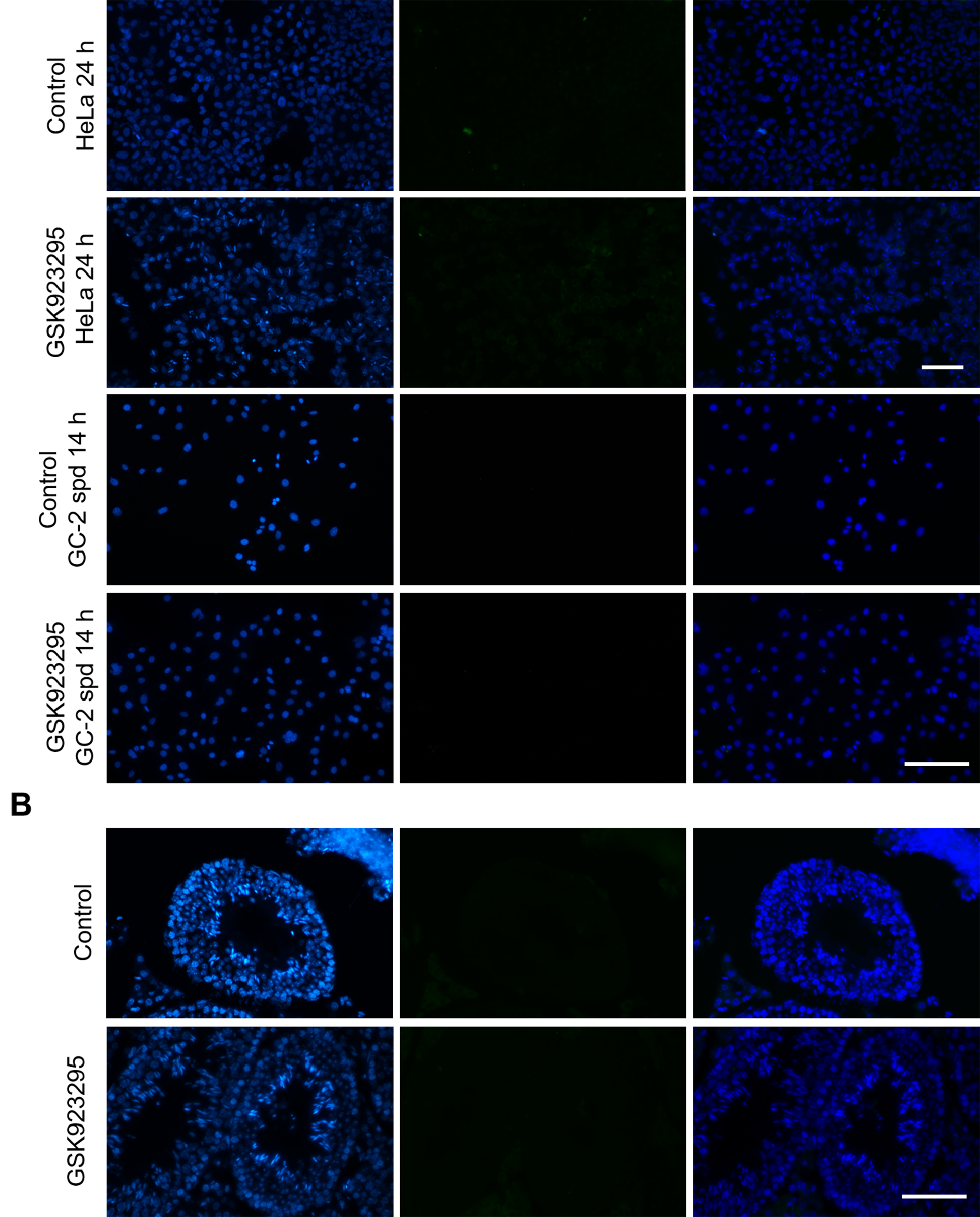

Supplement: Supplementary file 6 — Figure S6 [file 41420_2020_261_MOESM6_ESM.tif]
