# Supplementary material for: Kinesin-7 CENP-E regulates chromosome alignment and genome stability of spermatogenic cells
Source: Cell Death Discov. 2020 Apr 20;6:25. doi: 10.1038/s41420-020-0261-8 (PMC7171076; doi:10.1038/s41420-020-0261-8)
Supplement: Supplementary file 7 — Supplementary Figure Legends [file 41420_2020_261_MOESM7_ESM.docx]

**Supplementary Figure Legends**

**Kinesin-7 CENP-E Regulates Chromosome Alignment and Genome Stability of Spermatogenic Cells**

Zhen-Yu She, Kai-Wei Yu, Ning Zhong, Yu Xiao, Ya-Lan Wei, Yang Lin, Yue-Ling Li, Ming-Hui Lu

**Fig. S1.** The inhibition of CENP-E using specific inhibitor GSK923295, related to Fig. 1. **a** Representative images of mouse testes injected with 1 μM, 2 μM, 4 μM GSK923295. n = 4 per group (Control, 0.32 ± 0.02, n = 5; 1 μM, 0.29 ± 0.03, n = 4; 2 μM, 0.35 ± 0.03, n = 5; 4 μM, 0.27 ± 0.01, n = 4). **b** Ratios of testis weight/body weight of testis after injected with 1 μM and 2 μM, 4 μM GSK923295. Mean values ± SEM were shown. ns, p > 0.05. **c** Manchette had a wide range of rounding after CENP-E inhibition. **d** More lipid droplets appeared in the GSK923295 group compared with the control group. Scale bar, 5 μm. **e** DAPI (blue) and β-tubulin (green). Scale bar, 50 μm. There is no nonspecific fluorescence.

**Fig. S2.** Supplementary seminiferous tubules staging and seminiferous tubule deformity after CENP-E inhibition, related to Fig. 2. **a** Supplemental HE staining of mouse spermatogenesis in control group. Scale bar, 50 μm. **b** Additional seminiferous tubule malformations. Scale bar, 50 μm. The enlarged images were shown at the right panel. Scale bar, 20 μm.

**Fig. S3. CENP-E regulates chromosome alignment in HeLa cells.** Related to Fig.6. **a** Representative images of the HeLa cells in the control and GSK923295 groups. GSK923295 was added into the medium at a final concentration of 400 nM and cultured for 24 h. Scale bar, 100 μm. **b** Giemsa staining of Hela cells after incubated with 400 nM GSK923295 for 6 h and 24 h. Scale bar, 5 mm. **c** The ratios of arrested HeLa cells at metaphase after incubated with 400 nM GSK923295 for 24 h (Control, 7.20 ± 0.35%, group = 4, n = 426; GSK923295, 82.06 ± 3.66%, group = 4, n = 451). **d** Immunofluorescence of CENP-E in HeLa cells. DAPI (blue), CENP-E (green) and β-tubulin (red). Scale bar, 10 μm. **e** Giemsa staining of HeLa cells after incubated with 400 nM GSK923295 for 24 h. The cell cycle arrest (Scale bar, 50 μm), the huge cells (Scale bar, 20 μm) and the multinucleated cell (Scale bar, 5 μm). **f** Immunofluorescence of HeLa cells after incubated with 400 nM GSK923295 for 24 h. Each phenotype of the abnormal cells was shown. DAPI (blue) and β-tubulin (green). Scale bar, 5 μm. **g** The ratios of abnormal spindles, Control, 3.38 ± 2.20%, group = 3, n = 116; 3 h, 23.85 ± 6.66%, group = 3, n = 134; 6 h, 37.66 ± 3.36%, group = 3, n = 273; 24 h, 87.13 ± 0.93%, group = 3, n = 156, and the multipolar spindles (h), Control, 1.71 ± 0.86%, group = 3, n = 116; 3 h, 1.52 ± 1.52%, group = 3, n = 134; 6 h, 5.42 ± 1.76%, group = 3, n = 273; 24 h, 4.53 ± 0.79%, group = 3, n = 156.

**Fig. S4. Representative types of sperm abnormalities after CENP-E inhibition.** Related to Fig. 7. **a** Supplementary more comprehensive but small amount of deformed sperm. Scale bar, 20 μm.

**Fig. S5. The electronic microscopic images of control and GSK923295 treated group**. Related to Fig. 4. **a** The spermatids in the control and GSK923295 treated group. Scale bar, 5 μm. Arrows indicate the heterochromatin. **b** The primary spermatocytes arrested in metaphase. Scale bar, 5 μm. Arrows indicate the chromosomes.

**Fig. S6. Inhibition of CENP-E did not result in extensive apoptosis.** Related to Fig. 6. **a** Cell apoptosis of HeLa cells was detected using the TUNEL method after GSK923295 treatment. The apoptosis of GC-2 spd cells was detected using the TUNEL method at 14 hours after GSK923295 treatment. Scale bar, 100 μm. **b** The apoptosis of mouse testes was detected by TUNEL method after GSK923295 treatment. Scale bar, 100 μm.
